# Investigations of the distant metastatic non‐small cell lung cancer without local lymph node involvement: Real world data from a large database

**DOI:** 10.1111/crj.13668

**Published:** 2023-07-24

**Authors:** Bao‐Wen Huang, Wen‐Qin Wang, Jing‐Sheng Cai, Su‐Wen Zhang

**Affiliations:** ^1^ Department of Thoracic Surgery Sun Yat‐sen University Cancer Center Guangzhou China; ^2^ State Key Laboratory of Oncology in South China, Collaborative Innovation Center for Cancer Medicine Sun Yat‐sen University Cancer Center Guangzhou China; ^3^ Department of Thoracic Surgery Peking University People's Hospital Beijing China

**Keywords:** nomogram, non‐small cell lung cancer, prognosis, survival, T1‐4N0M1

## Abstract

**Introduction:**

This study aimed to investigate the presentations and survival outcomes of the distant metastatic non‐small cell lung cancer (NSCLC) without lymph node involvement to obtain a clearer picture of this special subgroup of metastatic NSCLC.

**Method:**

A least absolute shrinkage and selection operator (LASSO) penalized Cox regression analysis was used to select the prognostic variables. A nomogram and corresponding risk‐classifying systems were constructed. The C‐index and calibration curves were used to evaluate the performance of the model. Overall survival (OS) curves were plotted using the Kaplan–Meier method, and the log‐rank test was used to compare OS differences between groups. Propensity score matching (PSM) was performed to reduce bias.

**Result:**

A total of 12 610 NSCLC patients with M1 category (N0 group: 3045 cases; N1‐3 group: 9565 cases) were included. Regarding the N0 group, multivariate analysis demonstrated that age, sex, race, surgery, grade, tumor size, and M category were independent prognostic factors. A nomogram and corresponding risk‐classifying systems were formulated. Favorable validation results were obtained from the C‐index, calibration curves, and survival comparisons. Survival curves demonstrated that N0 NSCLC patients had better survival than N1‐3 NSCLC patients both before and after PSM. Furthermore, the survival of resected N0M1 patients was superior to that of those without surgery.

**Conclusion:**

In this study, a prognostic nomogram and risk‐classifying systems designed for the T1‐4N0M1 NSCLC patients showed acceptable performance. Primary lung tumor resection might be a feasible treatment for this population subset. Additionally, we proposed that lymph node stage might have a place in the forthcoming tumor‐node‐metastasis (TNM) staging proposal for NSCLC patients with M1 category.

## Graphical abstract

The central messages of this study are presented in this graphical abstract. NSCLC is non‐small cell lung cancer.

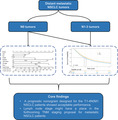

## 1 INTRODUCTION

Non‐small cell lung cancer (NSCLC) is one of the leading causes of cancer‐related mortality worldwide.^1^, ^2^, ^3^ Although the growing implementation of lung cancer screening has resulted in a dramatic increase in the early detection of NSCLC,^4^ in approximately 60% of NSCLC patients, local and distant metastases have already manifested by the time of initial diagnosis.^1^, ^3^ T1‐4N0M1 NSCLC, one subset of metastatic NSCLC, is defined as a tumor with local or distant metastases but with no regional lymph node involvement.^5^ It is widely acknowledged that NSCLC metastasis is a stepwise process. Regional lymph nodes are the most common metastatic sites in the initial stage, and then tumor cells can transfer to other parts of the body. Therefore, T1‐4N0M1 NSCLC is a special subgroup of metastatic lung cancer that needed further explorations.

To date, the number of related studies on this subset population is modest.^6^, ^7^ The unique epidemiologic characteristics and prognostic indicators of these patients have not been thoroughly investigated. In addition, as per the current TNM staging system, the TNM stage of metastatic NSCLC is only determined by M category (M1a and M1b are categorized as stage IVA; M1c is categorized as stage IVB).^5^ However, previous studies suggested that lymph node status may have an impact on the survival of these patients.^6^, ^7^ Thus, whether T1‐4N0M1 and T1‐4N1‐3M1 patients have homogenous clinical features and survival outcomes remains enigmatic.

In this study, we analyzed the data of the NSCLC cases with M1 category from the Surveillance, Epidemiology, and End Results (SEER) database to define the clinicopathological characteristics, prognosis, and survival of this population, with the purpose of obtaining a clearer picture of this subset of tumors.

## 2 MATERIALS AND METHODS

### 2.1 Patient Selection

Between 2010 and 2016, a series of 360 702 NSCLC cases were extracted from the SEER database by using SEER*Stat software version 8.3.4. Permission was obtained to retrieve SEER data files with the reference number: 12962‐Nov2019. Due to the fact that patient data in the SEER database are de‐identified, this study was dispensed with signing informed consent forms and acquiring ethical approval.

All included cases fit the following criteria: [1] confirmed as NSCLC; [2] diagnosed as M1 category (8th TNM staging system); and [3] received treatments. The exclusion criteria were as follows: [1] clinicopathological characteristics unknown; [2] age < 18 years old; [3] previous or concurrent other cancers; and [4] survival time ≤ 1 month.

**FIGURE 1:**
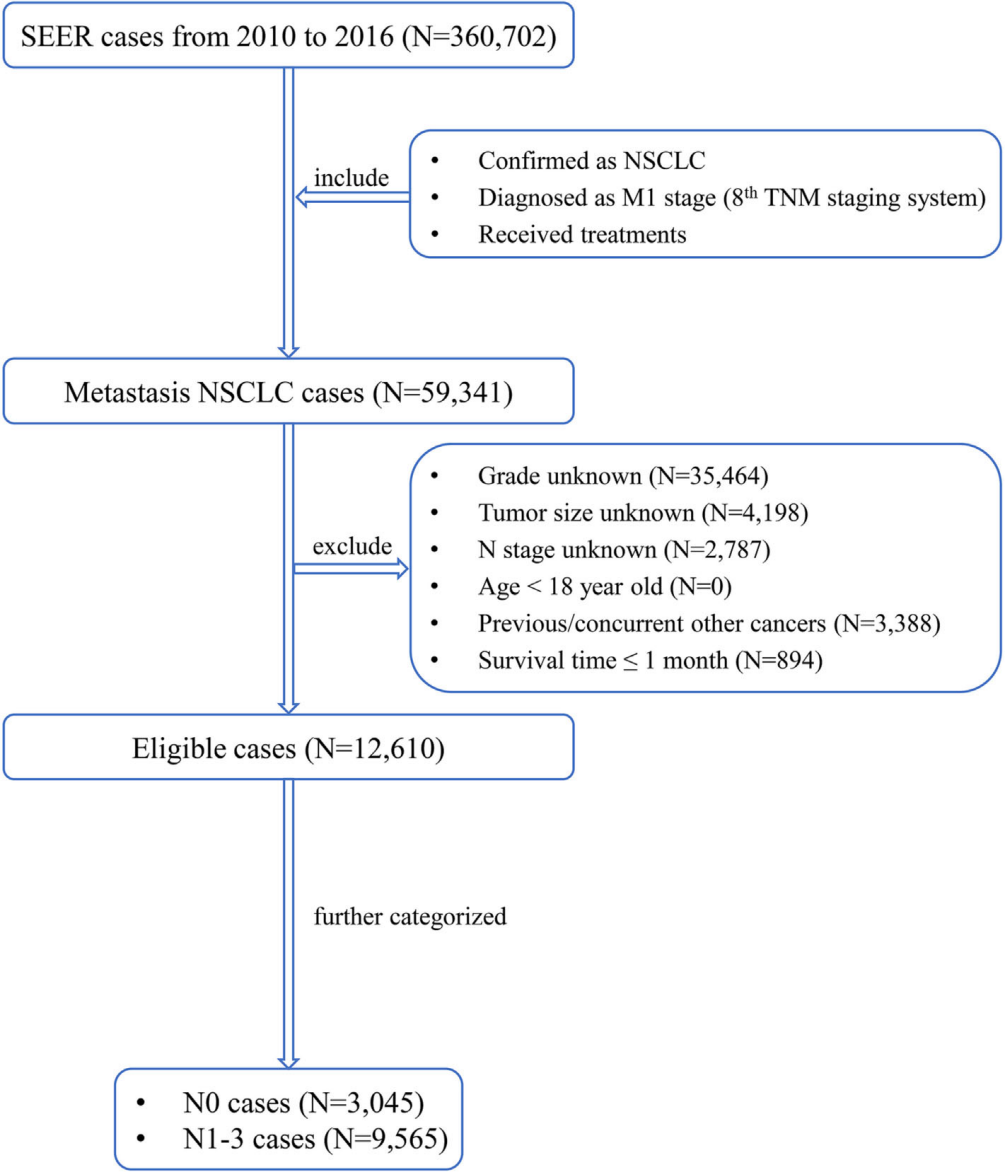
The study flow for patient selection. NSCLC, non‐small cell lung cancer; SEER, Surveillance, Epidemiology, and End Results; TNM, tumor‐node‐metastasis.

The entire cohort was categorized into two subgroups: the N0 group and the N1‐3 group. The N0 group was further divided into the training group and the validation group with a ratio of 3:1, and the development of the prognostic nomogram was based on data in the training set. The study flow for patient selection is depicted in Figure 1.

### 2.2 Data collection

The patients' demographic and clinicopathological features included sex (male and female), age (continuous form and categorical form: ≤60 years old and >60 years old), race (white, black, and other), marital status (married and other), tumor location (upper lobe, middle lobe, low lobe, and other), histology (adenocarcinoma, squamous cell carcinoma, and other), tumor size (continuous form and categorical form: 1–30 mm, 30–50 mm, 50–70 mm, and >70 mm), tumor grade (I: well differentiated; II: moderately differentiated; III: poor/undifferentiated), surgery (no and yes), radiotherapy (no and yes), chemotherapy (no and yes), M category (1a, 1b, and 1c), bone metastasis (no and yes), brain metastasis (no and yes), liver metastasis (no and yes), patient status, and survival time. A complete data analysis was performed in this study. The 6th and 7th editions of the TNM staging systems were converted to the current 8th edition of the TNM staging system. Because several patients have not received surgical resection, so both the pathological and clinical TNM stage was used in this study.

### 2.3 Follow‐up

Information on survival time and patient status was available in the SEER database. Patients who had definitive status and exact survival time were included in this study. Patients whose survival time was less than or equal to 1 month were excluded. Overall survival (OS), defined as the interval between the date of diagnosis and the date of death from any cause or the last follow‐up, was the primary endpoint of this study. The median follow‐up time was 10 months (range from 2 to 83 months) in the entire cohort, 13 months (range from 2 to 83 months) in the N0 group, and 10 months (range from 2 to 83 months) in the N1‐3 group.

### 2.4 Statistical analysis

Statistical analyses were carried out by R version 4.1.1 (The R Foundation for Statistical Computing, Vienna, Austria; http://www.r-project.org) and IBM SPSS Statistics (version 25.0, IBM Corp, Armonk, NY, USA). Least absolute shrinkage and selection operator (LASSO) regression analysis was performed to select and minimize the prognostic variables of the N0 group.^8^ Then, prognostic variables selected from the LASSO regression analysis were entered into a forward stepwise multivariable Cox proportional hazards regression model to determine the independent prognostic factors associated with mortality. A prognostic nomogram of N0 patients was developed based on the results of multivariable analysis.^9^ Harrell's C‐index^10^ and calibration curves were used to evaluate the performance of the nomogram. Receiver operating characteristic (ROC) curve with a corresponding area under curve (AUC) was used to compare the differences of predictive capability between risk classifying systems and TNM stage system. Each category of these prognostic variables was first assigned a score on the point scale in the nomogram. After summing the total score, X‐tile software^11^ was used to dichotomize the continuous score into two subgroups (low‐risk and high‐risk) and three subgroups (low‐risk, medium‐risk, and high‐risk). The Kaplan–Meier method was used to plot survival curves, and differences between survival curves were evaluated by the log‐rank test. A 1:1 propensity score matching (PSM) method was employed to reduce bias. Categorical variables, provided as frequencies and percentages, were compared using the Pearson *χ* ^2^ test between groups. Continuous variables, provided as the mean and standard deviation (SD), and median (range), were compared using the Mann–Whitney *U* test between groups. Two‐sided *P* values < 0.05 was considered statistically significant.

## 3 RESULTS

### 3.1 Patient characteristics

From 2010 to 2016, a series of 360 702 NSCLC cases from the SEER dataset were retrospectively evaluated. A total of 12 610 eligible M1 cases were retained following the application of our inclusion and exclusion criteria and were separated into the N0 group (*N* = 3045) and the N1‐3 group (*N* = 9565). The N0 group patients were randomly divided further into a training cohort (*N* = 2284) and a validation cohort (*N* = 761).

The general demographic, clinical, and pathological characteristics of the N0 and N1‐3 NSCLC cases are described in Table 1. With respect to the N0 NSCLC group, the median age was 66 years old (rang 18 to 100 years old), and there was no sexual predilection (50.7% vs. 49.3%). Over half of the cases were diagnosed with adenocarcinoma (ADC). There was a higher proportion of poor/undifferentiated tumors in this cohort (56.5%). Most patients had received chemotherapy rather than surgical resection and radiotherapy (87.4% vs. 23.0% vs. 17.4%). Approximately 51.8% of patients presented with only one distant metastatic lesion. In contrast to N1‐3 NSCLC patients, more patients in the N0 group were older (*P* < 0.001) and had smaller tumor sizes (*P* < 0.001). More poorly differentiated tumors were diagnosed in the N1‐3 NSCLC group than in the N0 group (*P* < 0.001). Patients in the N1‐3 NSCLC group were less likely to receive surgery (*P* < 0.001) and radiotherapy (*P* < 0.001), and almost all of the patients had received chemotherapy (96.3%, *P* < 0.001). The clinicopathological features of the patients with N0 and N1‐3 category after PSM were summarized in Table S1. The clinicopathological features of the N0 patients without surgery and with surgery after PSM were summarized in Table S2.

**TABLE 1:** The demographic, clinical, and pathological characteristics of the M1 NSCLC.

Characteristic	TxN0M1 NSCLC (*N* = 3045)	TxN1‐3M1 NSCLC (*N* = 9565)	*P*
Age, year			
Mean ± SD	65.8 ± 10.7	64.5 ± 10.6	<0.001^a^
Median (range)	66 (18–100)	65 (20–96)	
≤60	930 (30.5)	3389 (35.4)	<0.001
>60	2115 (69.5)	6176 (64.6)	
Sex			<0.001
Male	1545 (50.7)	5244 (54.8)	
Female	1500 (49.3)	4321 (45.2)	
Race			0.106
White	2391 (78.5)	7335 (76.7)	
Black	371 (12.2)	1251 (13.1)	
Other	283 (9.3)	979 (10.2)	
Marital status			0.249
Married	1687 (55.4)	5413 (56.6)	
Other	1358 (44.6)	4152 (43.4)	
Location			0.286
UL	1689 (55.5)	5432 (56.8)	
ML	137 (4.5)	412 (4.3)	
LL	893 (29.3)	2646 (27.7)	
Other	326 (10.7)	1075 (11.2)	
Histology			0.286
ADC	1767 (58.0)	5471 (57.2)	
SCC	631 (20.7)	2111 (22.1)	
Other	647 (21.2)	1983 (20.7)	
Tumor size (mm)			
Mean ± SD	45.6 ± 31.2	53.4 ± 39.5	<0.001^a^
Median (range)	40 (2–989)	48 (1–989)	
1–30	1027 (33.7)	2160 (22.6)	<0.001
30–50	1001 (32.9)	3079 (32.2)	
50–70	559 (18.4)	2223 (23.2)	
>70	458 (15.0)	2103 (22.0)	
Grade			<0.001
I	301 (9.9)	478 (5.0)	
II	1023 (33.6)	2685 (28.1)	
III	1721 (56.5)	6402 (66.9)	
Surgery			<0.001
No	2346 (77.0)	8901 (93.1)	
Yes	699 (23.0)	664 (6.9)	
Radiotherapy			<0.001
No	2514 (82.6)	8233 (86.1)	
Yes	531 (17.4)	1332 (13.9)	
Chemotherapy			<0.001
No	385 (12.6)	351 (3.7)	
Yes	2660 (87.4)	9214 (96.3)	
M stage			<0.001
1a	1145 (37.6)	2768 (28.9)	
1b	1576 (51.8)	4989 (52.2)	
1c	324 (10.6)	1808 (18.9)	
Bone metastasis			<0.001
No	2108 (69.2)	5506 (57.6)	
Yes	937 (30.8)	4059 (42.4)	
Brain metastasis			0.196
No	2085 (68.5)	6429 (67.2)	
Yes	960 (31.5)	3136 (32.8)	
Liver metastasis			<0.001
No	2682 (88.1)	7890 (82.5)	
Yes	363 (11.9)	1675 (17.5)	

Abbreviations: ADC, adenocarcinoma; LL, low lobe; ML, middle lobe; NSCLC, non‐small cell lung cancer; SCC, squamous cell carcinoma; SD, standard deviation; UL, upper lobe.

^a^ Mann–Whitney *U* test.

### 3.2 LASSO penalized multivariate Cox regression analysis

**FIGURE 2:**
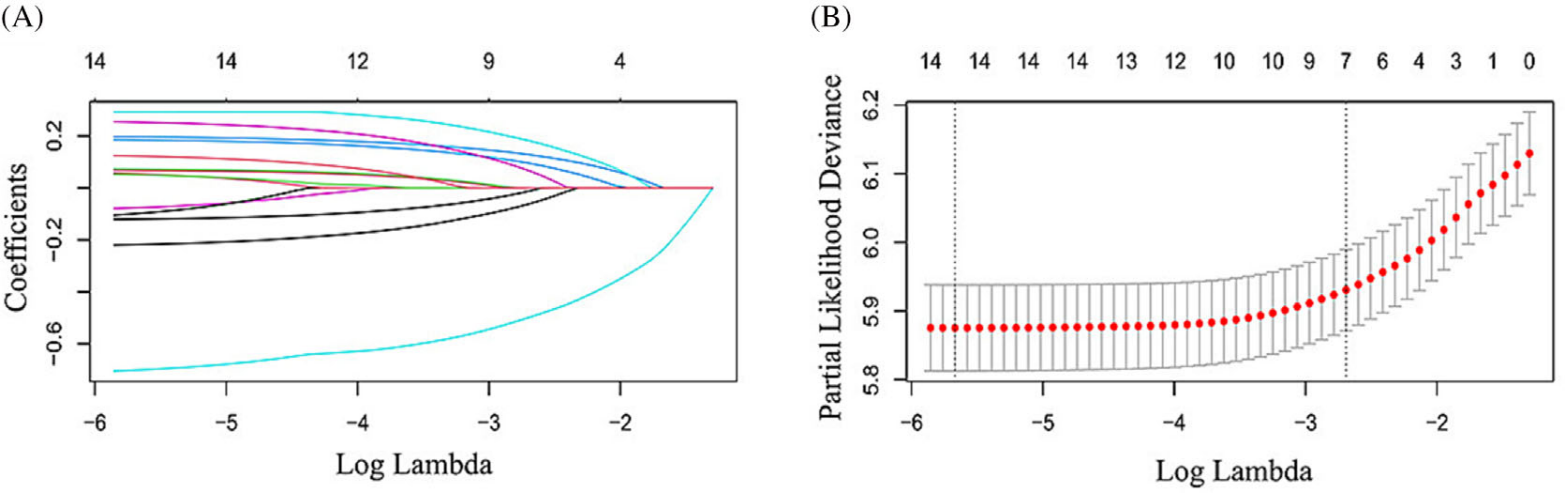
Prognostic variables selection using the LASSO model analysis. LASSO coefficient profiles of 15 covariates against the log (Lambda) sequence for OS (A). tuning parameter (Lambda) selection in the LASSO model used 10‐fold cross‐validation via minimum criteria for OS (B). LASSO, least absolute shrinkage and selection operator; OS, overall survival.

**FIGURE 3:**
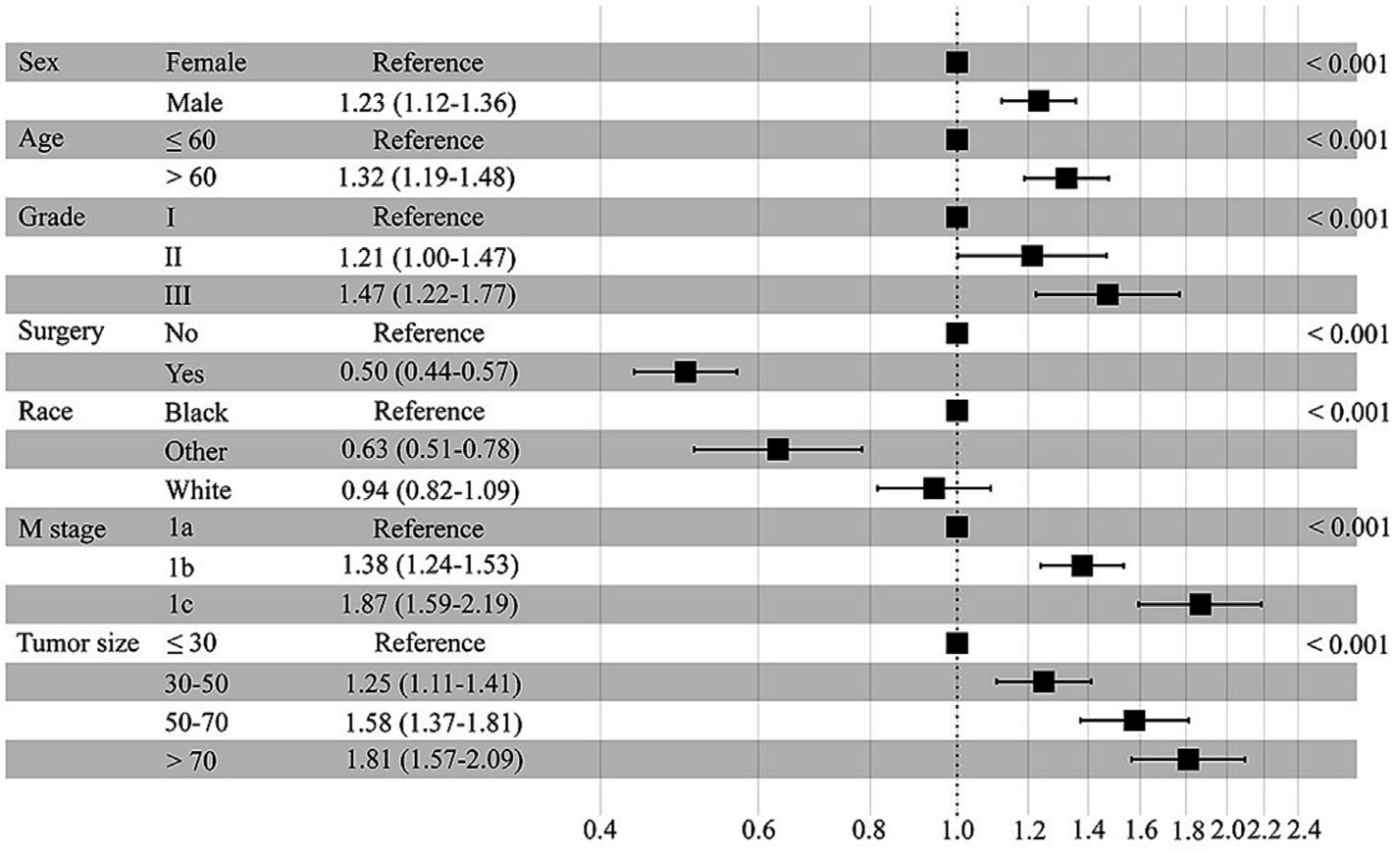
The forest plot of the multivariate Cox regression model.

LASSO regression model analysis was carried out to select the prognostic factors of N0 NSCLC with the best predictive performance using 10‐fold cross‐validation in the training set^8^ (Figure 2). Fifteen factors, including age, sex, race, marital status, location, surgery, radiotherapy, chemotherapy, histology, grade, tumor size, M stage, bone metastasis, brain metastasis, and liver metastasis, were entered into the LASSO regression analysis, and only seven factors, including age, sex, race, surgery, grade, tumor size, and M stage, were entered into the multivariable Cox analysis. The multivariable Cox analysis confirmed that age (HR = 1.32, 95% CI: 1.19–1.48, *P* < 0.001), sex (HR = 1.23, 95% CI: 1.12–1.36, *P* < 0.001), race (HR = 0.63, 95% CI: 0.51–0.78, *P* < 0.001), surgery (HR = 0.50, 95% CI: 0.44–0.57, *P* < 0.001), grade (HR = 1.47, 95% CI: 1.22–1. 77, *P* < 0.001), tumor size (HR = 1.81, 95% CI: 1.57–2.09, *P* < 0.001), and M stage (HR = 1.87, 95% CI: 1.59–2.19, *P* < 0.001) were independent prognostic factors (Figure 3). A LASSO penalized multivariable Cox analysis was conducted in N1‐3 patients, and the results showed that in addition to the prognostic factors mentioned in N0 patients, N1‐3 patients also benefited from chemotherapy (HR = 0.509, 95% CI: 0.451–0.575, *P* < 0.001).

### 3.3 Nomogram and validation

**FIGURE 4:**
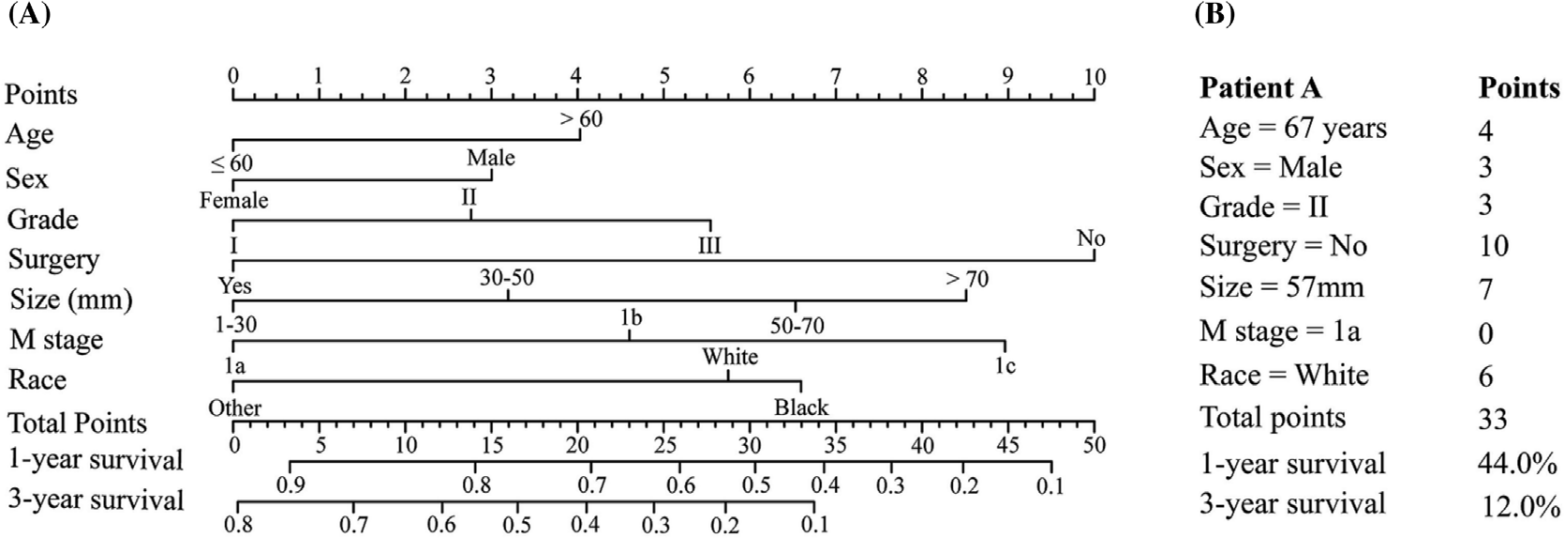
Nomogram for predicting the 1‐year and 3‐year OS in N0M1 non‐small cell lung cancer (NSCLC) patients (A). Each number or category of these prognostic factors is assigned a score on the Points scale. After summing up the total score of all the prognostic factors and locating it on the Total Points scale, a line drawn straight down to 1‐/3‐year survival probability scale shows the estimated survival probability at each time point. We have used this nomogram to estimate a patient's survival (B). OS, overall survival.

Based on the results of multivariate Cox analysis, a prognostic nomogram was constructed (Figure 4A). The nomogram illustrated that surgery had the largest contribution to prognosis, followed by M stage, tumor size, and race. The C‐indexes of the training cohort and the validation cohort were 0.66 (95% CI: 0.65–0.67) and 0.66 (95% CI: 0.63–0.69), respectively. The calibration plots for the probability of 1‐year and 3‐year survival were all in good agreement (Figure S1). Furthermore, this nomogram was used to estimate a patient’ s survival (Figure 4B).

### 3.4 Evaluation of risk‐classifying system

**FIGURE 5:**
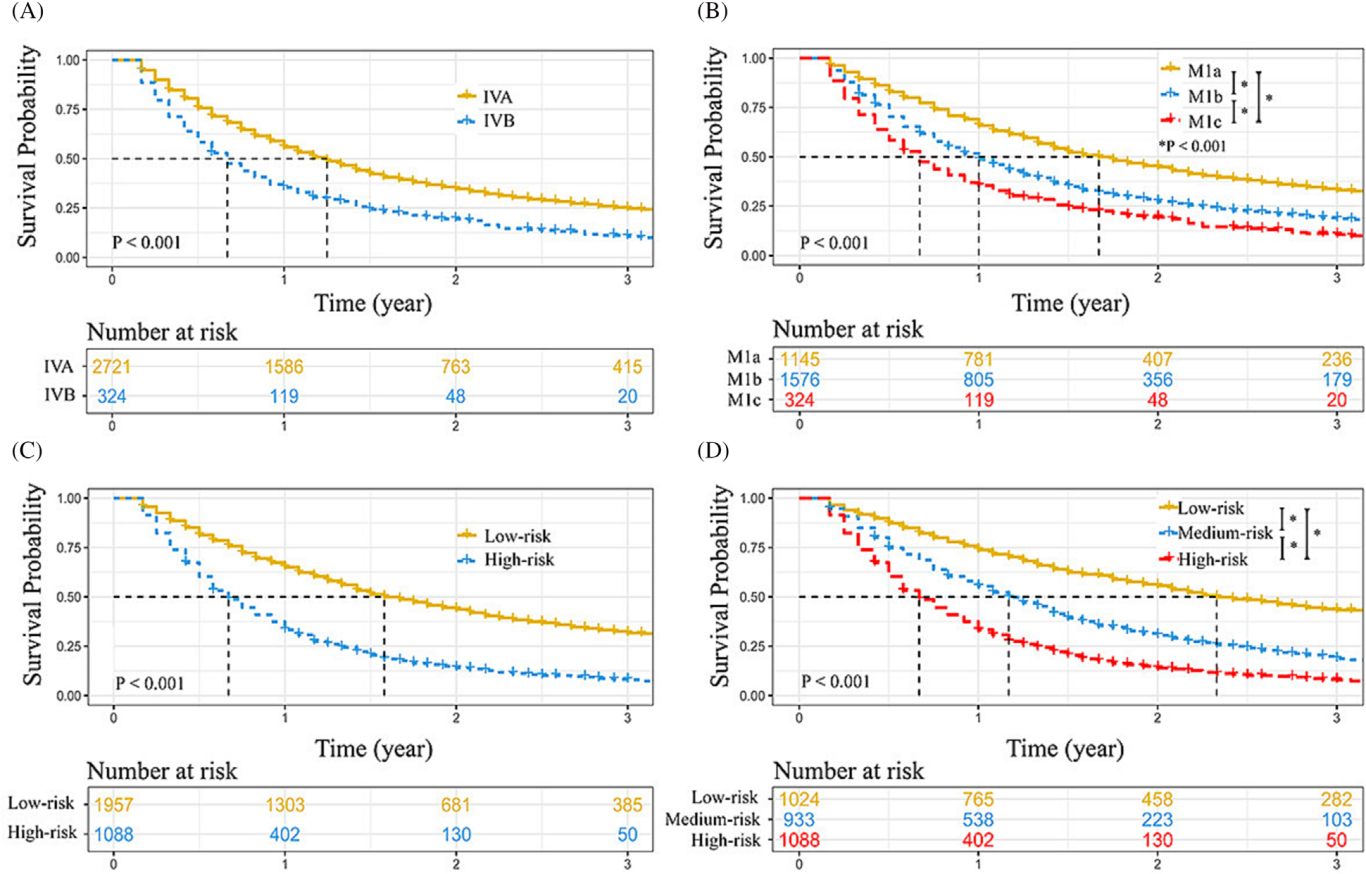
Kaplan–Meier curve comparisons. (A) stage IVA vs. stage IVB, (B) M1a vs. M1b vs. M1c, (C) low‐risk vs. High‐risk, and (D) low‐risk vs. medium‐risk vs. high‐risk.

Each category of the prognostic factors was assigned a score on the points scale in the nomogram. After summing the total score, the continuous scores were divided into two subgroups (low‐risk and high‐risk, risk‐classifying system A) based on the cutoff value (33) and into three subgroups (low‐risk, medium‐risk, and high‐risk, risk‐classifying system B) based on the cutoff values (26 and 33) (Table 2). According to the TNM stage, the N0 NSCLCs were also assigned into two subgroups (stage IVA and stage IVB, TNM staging system) and three subgroups (M1a, M1b, and M1c, M staging system). The survival curves demonstrated that the OS of stage IVA patients was superior to that of stage IVB patients (HR: stage IVA vs. stage IVB = 1 vs. 1.707, 95% CI: 1.507–1.933, *P* < 0.001, Figure 5A), and a stepwise deterioration of OS was observed with the increase in M stage (HR: M1a vs. M1b vs. M1c = 1 vs. 1.564 vs. 2.213, *P* < 0.001, Figure 5B). Our risk‐classifying system A demonstrated that the low‐risk patients had better OS than the high‐risk patients (HR: low‐risk vs. high‐risk = 1 vs. 2.287, 95% CI: 2.102–2.489, Figure 5C), and a progressive degradation of OS with the increase in risk was also shown in risk‐classifying system B (HR: low‐risk vs. medium‐risk vs. high‐risk = 1 vs. 1.983 vs. 3.212, *P* < 0.001, Figure 5D). The results of ROC showed that the AUC of the risk‐classifying system A was superior than the TNM staging system (0.64 vs. 0.54, Figure S2A), and the AUC of the risk‐classifying system B was also superior than the M staging system (0.70 vs. 0.59, Figure S2C). DCA further confirmed that our risk‐classifying system A had a satisfying clinical net benefit when compared with the TNM staging system (Figure S2B), and patients could gain more benefits from the risk‐classifying system B when compared with the M staging system (Figure S2D).

**TABLE 2:** Prognostic score of each variable calculated based on the nomogram.

Characteristics	Category	Score
Age	≤60	0
	>60	4
Sex	Male	3
	Female	0
Grade	I	0
	II	3
	III	6
Surgery	No	10
	Yes	0
Tumor size	1–30	0
	30–50	3
	50–70	7
	>70	9
M stage	1a	0
	1b	5
	1c	9
Race	White	6
	Black	7
	Other	0
Risk‐classifying system (2 groups, cutoff value = 33)		
Low‐risk	≤33	
High‐risk	>33	
Risk‐classifying system (3 groups, cutoff value 1 = 26, cutoff value 2 = 33)		
Low‐risk	≤26	
Medium‐risk	26–33	
High‐risk	>33	

### 3.5 Survival analysis

**FIGURE 6:**
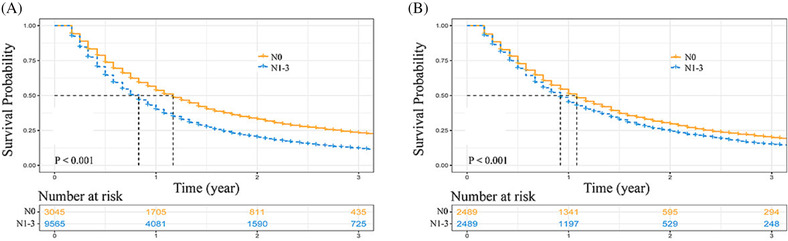
Kaplan–Meier curve comparisons. (A) Before propensity score matching (PSM): N0 vs. N1‐3; and (B) after PSM: N0 vs. N1‐3.

Before PSM, compared with N1‐3 NCSLC patients, N0 NSCLC patients had superior OS (3‐year OS rate: N0 vs. N1–3 = 23.2% vs. 12.1%, *P* < 0.001, Figure 6A). After PSM, N0 patients still enjoyed a higher level of survival than N1‐3 patients (3‐year OS rate: N0 vs. N1‐3 = 19.9% vs. 15.1%, *P* < 0.001, Figure 6B).

**FIGURE 7:**
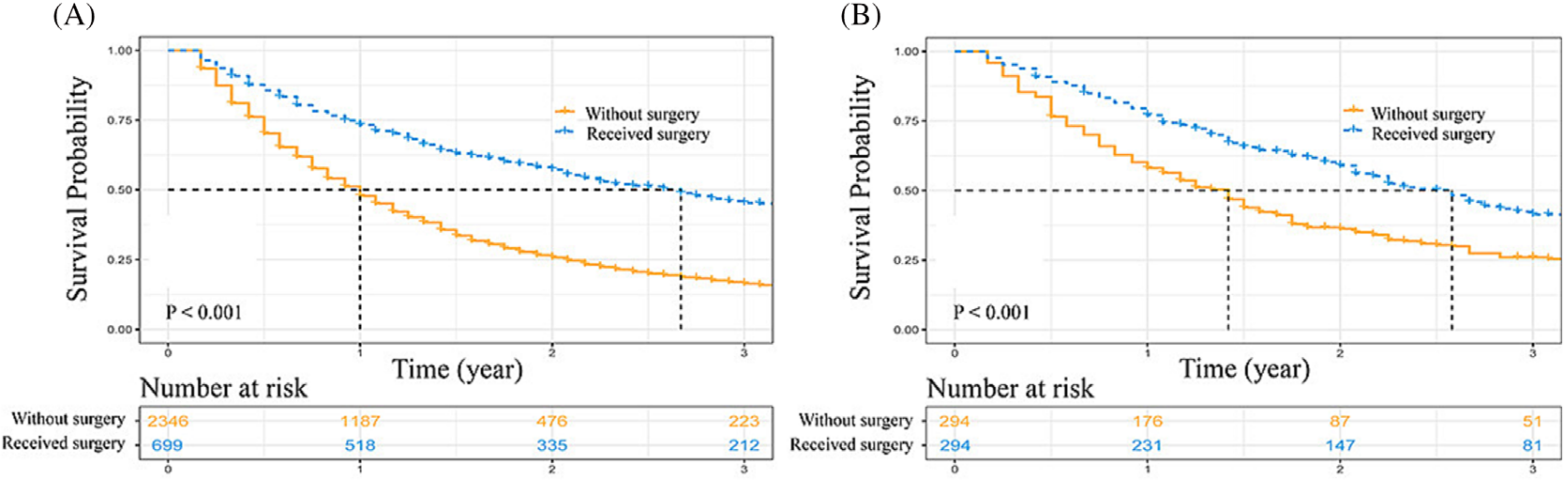
Kaplan–Meier curve comparisons. (A) Before PSM: Without surgery vs. Received surgery; and (B) after PSM: Without surgery vs. Received surgery. PSM, propensity score matching.

To further confirm that the N0 patients could benefit from surgical resection, survival comparisons were conducted between patients without surgery and patients with surgery. Before PSM, patients who underwent surgery had better OS than patients who did not undergo surgery (3‐year OS rate: without surgery vs. with surgery = 16.4% vs. 45.3%, *P* < 0.001, Figure 7A). After PSM, surgery still provided great survival benefit to these patients (3‐year OS rate: without surgery vs. with surgery = 26.0% vs. 41.4%, *P* < 0.001, Figure 7B).

## 4 DISCUSSION

In this study, we developed an individualized nomogram and corresponding risk‐classifying system including routinely available prognostic factors that were selected from a LASSO penalized multivariable Cox proportional hazards regression model to predict OS in a large cohort of T1‐4N0M1 NSCLC patients. Favorable validation results were obtained from C‐index, calibration curves, and survival comparisons. With the help of this nomogram, physicians may be able to predict the individual survival of T1‐4N0M1 NSCLC patients using this highly cost‐efficient scoring system. Additionally, we also revealed that the survival of N0M1 NSCLC was better than that of N1‐3M1 NSCLC that suggested that lymph node metastasis might play a critical role in the prognosis of M1 NSCLC.^12^

To our knowledge, no one has constructed a prognostic nomogram for this special metastatic NSCLC subset, and to date, more attention has been focused on metastasis site‐specific NSCLCs.^13^, ^14^, ^15^ The nomogram, an efficient statistical predictive model with a graphic representation, is able to integrate multiple prognostic indicators and decode the probability of an event more precisely than traditional evaluation standards.^16^ Regarding the current TNM staging system, the factors adopted to subdivide N0M1 NSCLC are metastasis sites and the number of involved organs.^5^ From our perspective, given the dismal survival rate of M1 NSCLC, more prognostic factors apart from the variables of the TNM staging system should be included when predicting the survival of these patients. In this study, our nomogram confirmed that the M stage was a stronger predictor. In addition, the nomogram indicated that receiving surgery, smaller tumor size, other races, high differentiation grade, younger age, and female sex were favorable factors. The inclusion of these additional factors could contribute to the superior power of our nomogram in predicting prognosis. Although the C‐index of our nomogram was not exceptional, greater accuracy of the model is usually accompanied by a dilemma between the increasing complexity of predictive factors and the decreasing utility of the model in clinical practice.^17^ Considering the aforementioned factors, variables of clinical importance and highly repeatable practicability would be preferred.

Based on our nomogram, two risk‐classifying systems were established. Favorable validation results suggested that our risk‐classifying system may be a useful tool that could estimate individual survival and help clinical decision making. These findings are important for clinical practice. Our risk‐classifying systems could offer clinicians quantitative tools to evaluate individual risk profiles more precisely that may help to expediently deliver personalized medical care and follow‐up strategies. More specifically, clinicians could calculate the risk score of each N0M1 patient and assign them to different risk groups. High‐risk patients may need more aggressive or novel therapeutic agents, such as immunotherapy to prolong their lives. It is likely that our results could play a supplementary role to the TNM staging system and aid in identifying members of a high‐risk population.

Our results implied that patients with N0M1 NSCLC who underwent surgical resection of primary lung lesions enjoyed high levels of survival when compared with those who did not.^18^ In line with our results, Xu et al. analyzed the data of 6466 stage IV NSCLC patients and demonstrated that the OS of NSCLC patients with primary tumor resection was superior to that of those without resection (27 vs. 8 months).^19^ A similar scenario was also observed in the studies of Asamura et al.^18^ and Strand et al.^20^ In our view, resection of primary lesions could alleviate tumor‐related symptoms and further improve patients' quality of life, which may extend patients' lives. Therefore, we suggest that although primary tumor resection is not recommended in the current guideline,^21^ surgery is still a feasible treatment choice for M1 patients whose systemic condition is stable.

In this study, we revealed that the survival of N0M1 NSCLC was better than that of N1‐3M1 NSCLC.^12^ Our results were similar to those of Yang et al., who demonstrated that as for stage IV NSCLC patients, lymph node metastasis was correlated with higher odds of multiple organ metastasis and a worse prognosis.^7^ In the study by Dai et al., the authors reviewed 39 731 M1a NSCLC patients from the SEER database and showed that the cancer‐specific survival of N0 disease was better than that of N1 disease and that the survival of N1 disease was better than that of N2 disease.^6^ The prognostic significance of tumor burden is also emphasized by several researchers.^22^, ^23^ In the current TNM staging system however, lymph node status is not incorporated into the final TNM stage.^5^ We believed that it is advisable to incorporate N stage into the final TNM staging system in order to achieve a more accurate prognostic stratification. Herein, we proposed that lymph node stage might have a place in the forthcoming 9th TNM staging proposal for M1 NSCLC patients. This gave us a hit, but more verifications are needed in the future. In addition, we also found that when compared with N0 patients, N1‐3 patients could benefit from chemotherapy. A possible explanation for this difference might be that anti‐tumor benefits far outweigh the adverse side‐effects in N1‐3 patients. So, we proposed that for N1‐3 patients, although these patients might be suffered from side effects, they still should receive chemotherapy as far as possible.

Despite the significant advantage provided by the large sample data from the SEER dataset, there are limitations to our report. First, this study focused on the distant metastatic NSCLC patients without lymph node involvements. Therefore, an accurate N category is a guarantee for obtaining reliable conclusions, and more powerful staging tools such as positron emission tomography‐computed tomography (PET‐CT), endobronchial ultrasound‐guided transbronchial needle aspiration (EBUS‐TBNA), and mediastinoscopy are needed. However, the related data are unavailable in the SEER database, and we hoped other data including the distant metastatic NSCLC patients with definite N category could validate our results in the future. Second, the SEER database also lacks data on novel therapies, the treatment schedule and the reason why these metastatic patients received surgery, and all these variables are important factors in survival analysis. Third, the patient population used to formulate this nomogram was predominantly of White ethnicity, and external validation from the Asian population is warranted. At last, although large sample data from the SEER database might be less prone to the bias toward the null hypothesis, selection bias may still be present due to the retrospective design of the study. Efforts to include prospective study designs and broader clinicopathological variables are encouraged for future studies.

## 5 CONCLUSION

In conclusion, in this study, an efficient nomogram and risk‐classifying systems were designed for T1‐4N0M1 NSCLC that showed acceptable performance. Primary lung tumor resection may be a feasible treatment for this population subset. In addition, we proposed that lymph node stage might have a place in the TNM staging proposal for the M1 NSCLC patients.

## AUTHOR CONTRIBUTIONS

(I) Conception and design: Su‐Wen Zhang and Jing‐Sheng Cai. (II) Administrative support: Su‐Wen Zhang. (III) Provision of study materials or patients: Bao‐Wen Huang and Wen‐Qin Wang. (IV) Collection and assembly of data: Bao‐Wen Huang. (V) Data analysis and interpretation: Bao‐Wen Huang. (VI) Manuscript writing: all authors. (VII) Final approval of manuscript: all authors.

## CONFLICT OF INTEREST STATEMENT

The authors declare that there is no conflict of interest regarding the publication of this article.

## ETHICS APPROVAL AND CONSENT TO PARTICIPATE

Permission was obtained to retrieve SEER data files with the reference number: 12962‐Nov2019. Due to the fact that patient data in the SEER database is de‐identified, this study was dispensed with signing informed consent forms and acquiring ethical approval.

## Supporting information


**Figure S1.** The calibration curves for predicting OS in the training cohort (A, B) and the validation cohort (C, D). Nomogram‐predicted survival probability is plotted on the x‐axis; Actual observed survival probability is plotted on the y‐axis. A curve along the 45‐degree line indicates perfect calibration models. OS, overall survival.

**Figure S2.** Validation of the risk‐classifying systems. (A) ROC curves comparison: Risk‐classifying system A vs. TNM staging system, (B) DCA comparison: Risk‐classifying system A vs. TNM staging system, (C) ROC curves comparison: Risk‐classifying system B vs. M staging system and (D) DCA comparison: Risk‐classifying system B vs. M staging system. ROC: receiver operating characteristic, DCA: decision curve analyses; TNM, tumor‐node‐metastasis

**Table S1.** The Clinical characteristics of the N0M1 and N1–3M1 patients after PSM
**Table S2.** The Clinical characteristics of the N0M1 patients without surgery and with surgery after PSM

## Data Availability

The dataset supporting the conclusions of this article is available in the SEER database (https://seer.cancer.gov/) that is available for the researchers who are authenticated.
