# Supplementary figures and images for: Investigations of the distant metastatic non‐small cell lung cancer without local lymph node involvement: Real world data from a large database
Source: Clin Respir J. 2023 Jul 24;17(8):780–90. doi: 10.1111/crj.13668 (PMC10435941; doi:10.1111/crj.13668)

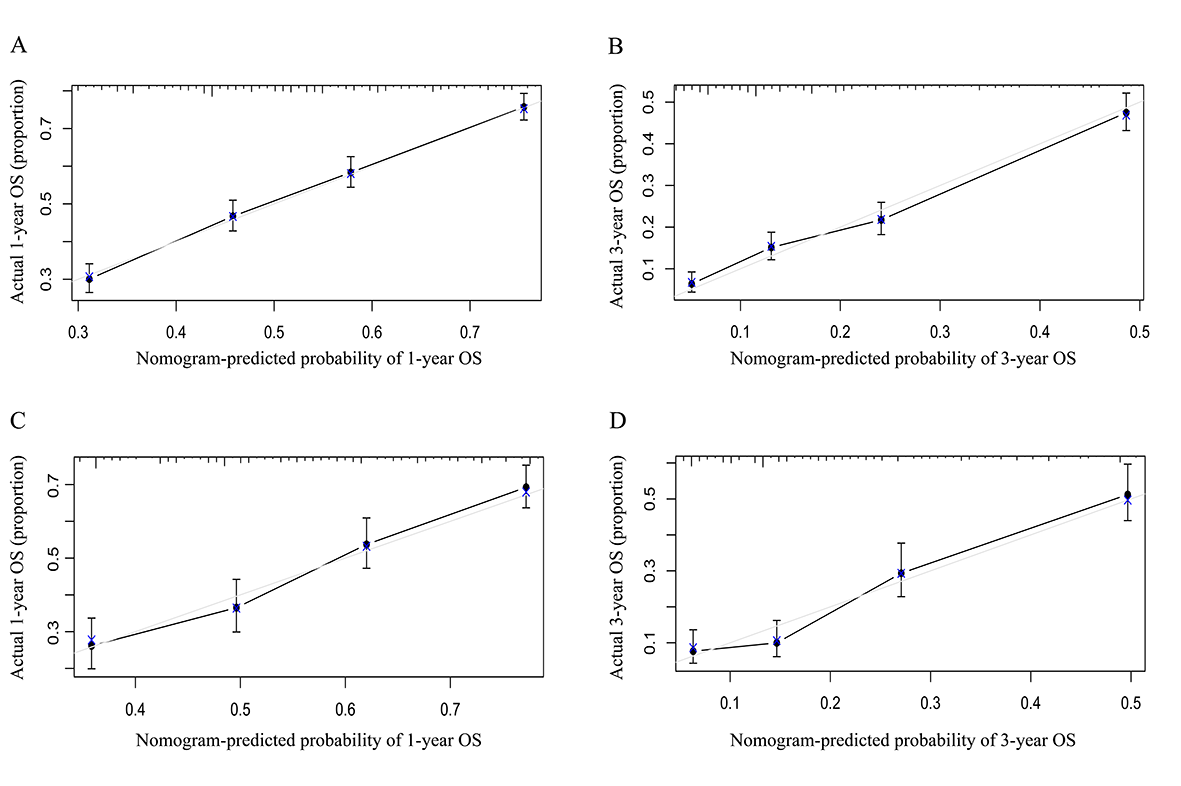

Supplement: Supplementary file 1 — Figure S1. The calibration curves for predicting OS in the training cohort (A, B) and the validation cohort (C, D). Nomogram‐predicted survival probability is plotted on the x‐axis; Actual observed survival probability is plotted on the y‐axis. A curve along the 45‐degree line indicates perfect calibration models. OS, overall survival. [file CRJ-17-780-s003.tif]

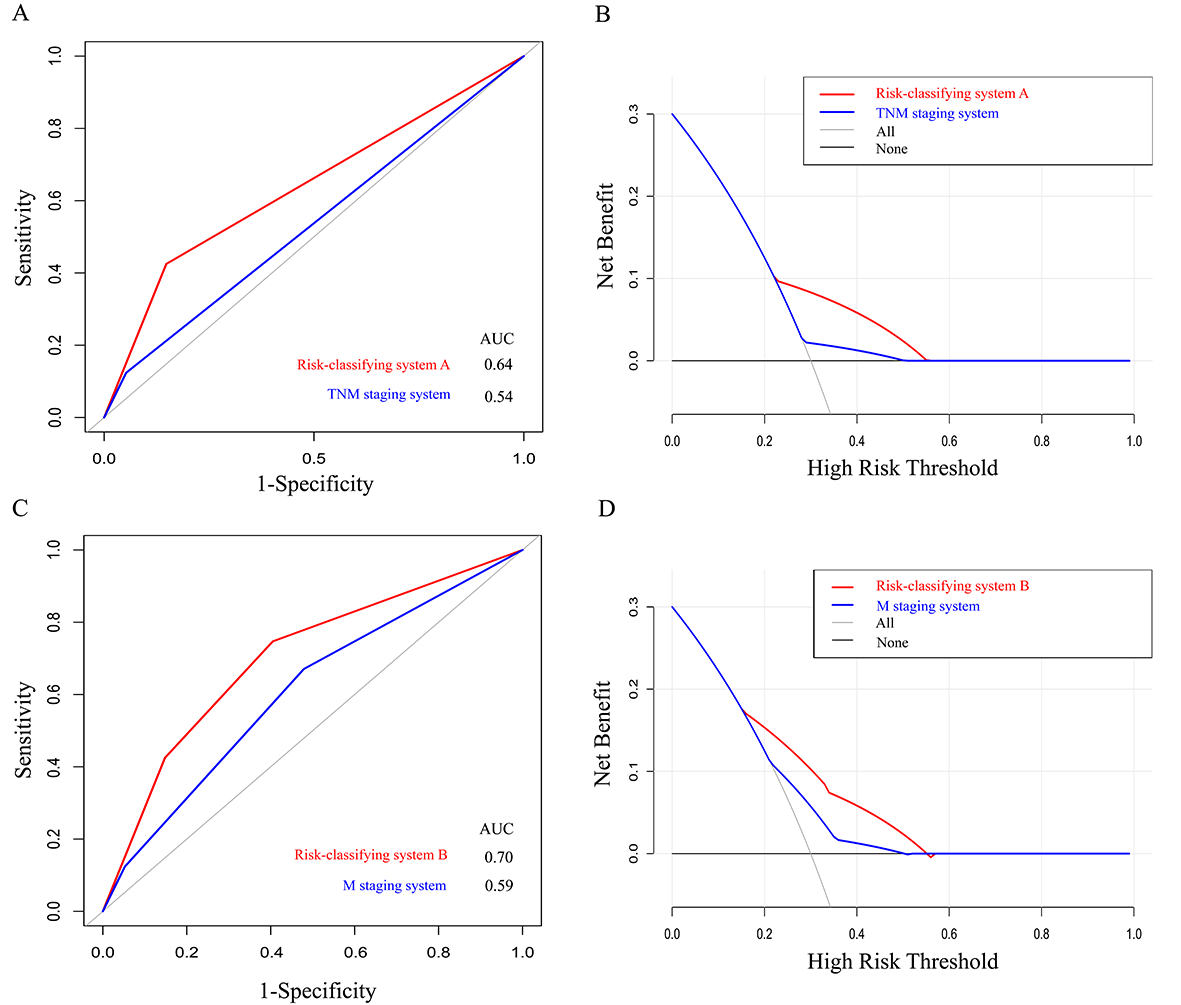

Supplement: Supplementary file 2 — Figure S2. Validation of the risk‐classifying systems. (A) ROC curves comparison: Risk‐classifying system A vs. TNM staging system, (B) DCA comparison: Risk‐classifying system A vs. TNM staging system, (C) ROC curves comparison: Risk‐classifying system B vs. M staging system and (D) DCA comparison: Risk‐classifying system B vs. M staging system. ROC: receiver operating characteristic, DCA: decision curve analyses; TNM, tumor‐node‐metastasis [file CRJ-17-780-s002.tif]
