# Supplementary material for: Investigations of the distant metastatic non‐small cell lung cancer without local lymph node involvement: Real world data from a large database
Source: Clin Respir J. 2023 Jul 24;17(8):780–90. doi: 10.1111/crj.13668 (PMC10435941; doi:10.1111/crj.13668)
Supplement: Supplementary file 3 — Table S1. The Clinical characteristics of the N0M1 and N1–3M1 patients after PSM Table S2. The Clinical characteristics of the N0M1 patients without surgery and with surgery after PSM [file CRJ-17-780-s001.docx]

**Table S1. The Clinical characteristics of the N0M1 and N1-3M1 patients after PSM**

| Characteristic | N0M1 | N1-3M1 | P |
| --- | --- | --- | --- |
| Age, year |  |  | 1.000 |
| ≤ 60 | 732 (29.4) | 732 (29.4) |  |
| > 60 | 1,757 (70.6) | 1,757 (70.6) |  |
| Sex |  |  | 0.799 |
| Male | 1,279 (51.4) | 1,288 (51.7) |  |
| Female | 1,210 (48.6) | 1,201 (48.3) |  |
| Race |  |  | 0.162 |
| White | 1,887 (75.8) | 1,735 (69.7) |  |
| Black | 351 (14.1) | 381 (15.3) |  |
| Other | 251 (10.1) | 37 (15.0) |  |
| Marital status |  |  | 0.749 |
| Married | 1,264 (50.7) | 1,277 (51.3) |  |
| Other | 1,225 (49.3) | 1,212 (48.7) |  |
| Location |  |  | 0.467 |
| UL | 1,388 (55.8) | 1,405 (56.4) |  |
| ML | 108 (4.3) | 128 (5.1) |  |
| LL | 722 (29.0) | 691 (27.8) |  |
| Other | 271 (10.9) | 265 (10.6) |  |
| Histology |  |  | 0.965 |
| ADC | 1,516 (60.9) | 1,524 (61.2) |  |
| SCC | 505 (20.3) | 498 (20.0) |  |
| Other | 468 (18.8) | 467 (18.8) |  |
| Tumor size (mm) |  |  | 0.984 |
| 1-30 | 734 (29.5) | 743 (29.9) |  |
| 30-50 | 890 (35.8) | 890 (35.8) |  |
| 50-70 | 476 (19.1) | 476 (19.1) |  |
| > 70 | 389 (15.6) | 380 (15.3) |  |
| Grade |  |  | 0.979 |
| I | 182 (7.3) | 180 (7.2) |  |
| II | 811 (32.6) | 806 (32.4) |  |
| III | 1,496 (60.1) | 1,503 (60.4) |  |
| Surgery |  |  | 1.000 |
| No | 2,207 (88.7) | 2,207 (88.7) |  |
| Yes | 282 (11.3) | 282 (11.3) |  |
| Radiotherapy |  |  | 0.699 |
| No | 2,183 (87.7) | 2,174 (87.3) |  |
| Yes | 306 (12.3) | 315 (12.7) |  |
| Chemotherapy |  |  | 1.000 |
| No | 107 (4.3) | 107 (4.3) |  |
| Yes | 2,382 (95.7) | 2,382 (95.7) |  |
| M stage |  |  | 0.963 |
| 1a | 899 (36.1) | 890 (35.8) |  |
| 1b | 1,324 (53.2) | 1,333 (53.6) |  |
| 1c | 266 (10.7) | 266 (10.7) |  |
| Bone metastasis |  |  | 1.000 |
| No | 1,652 (66.4) | 1,652 (66.4) |  |
| Yes | 837 (33.6) | 837 (33.6) |  |
| Brain metastasis |  |  | 0.782 |
| No | 1,732 (69.6) | 1,723 (69.2) |  |
| Yes | 757 (30.4) | 766 (30.8) |  |
| Liver metastasis |  |  | 1.000 |
| No | 2,201 (88.4) | 2,201 (88.4) |  |
| Yes | 288 (11.6) | 288 (11.6) |  |

UL, upper lobe; ML, middle lobe; LL, low lobe; ADC, adenocarcinoma; SCC, squamous cell carcinoma; NSCLC, non-small cell lung cancer; PSM, propensity score matching

**Table S2. The Clinical characteristics of the N0M1 patients without surgery and with surgery after PSM**

| Characteristic | Without surgery | With surgery | P |
| --- | --- | --- | --- |
| Age, year |  |  | 0.610 |
| ≤ 60 | 114 (38.8) | 108 (36.7) |  |
| > 60 | 180 (61.2) | 186 (63.3) |  |
| Sex |  |  | 0.804 |
| Male | 161 (54.8) | 158 (53.7) |  |
| Female | 133 (45.2) | 136 (46.3) |  |
| Race |  |  | 0.583 |
| White | 213 (72.4) | 224 (76.2) |  |
| Black | 46 (15.6) | 40 (13.6) |  |
| Other | 35 (11.9) | 30 (10.2) |  |
| Marital status |  |  | 0.406 |
| Married | 160 (54.4) | 170 (57.8) |  |
| Other | 134 (45.6) | 124 (42.2) |  |
| Location |  |  | 0.630 |
| UL | 159 (54.1) | 164 (55.8) |  |
| ML | 11 (3.7) | 16 (5.4) |  |
| LL | 84 (28.6) | 81 (27.6) |  |
| Other | 40 (13.6) | 33 (11.2) |  |
| Histology |  |  | 0.916 |
| ADC | 199 (67.7) | 195 (66.3) |  |
| SCC | 38 (12.9) | 38 (12.9) |  |
| Other | 57 (19.4) | 61 (20.7) |  |
| Tumor size (mm) |  |  | 0.955 |
| 1-30 | 142 (48.3) | 145 (49.3) |  |
| 30-50 | 88 (29.9) | 83 (28.2) |  |
| 50-70 | 31 (10.3) | 30 (10.2) |  |
| > 70 | 33 (11.2) | 36 (12.2) |  |
| Grade |  |  | 0.990 |
| I | 35 (11.9) | 34 (11.6) |  |
| II | 89 (30.3) | 90 (30.6) |  |
| III | 170 (57.8) | 170 (57.8) |  |
| Radiotherapy |  |  | 0.932 |
| No | 186 (63.3) | 187 (63.6) |  |
| Yes | 108 (36.7) | 107 (36.4) |  |
| Chemotherapy |  |  | 0.759 |
| No | 22 (7.5) | 24 (8.2) |  |
| Yes | 272 (92.5) | 270 (91.8) |  |
| M stage |  |  | 0.956 |
| 1a | 144 (49.0) | 146 (49.7) |  |
| 1b | 139 (47.3) | 136 (46.3) |  |
| 1c | 11 (3.7) | 12 (4.1) |  |
| Bone metastasis |  |  | 0.500 |
| No | 250 (85.0) | 244 (83.0) |  |
| Yes | 44 (15.0) | 50 (17.0) |  |
| Brain metastasis |  |  | 0.536 |
| No | 196 (66.7) | 203 (69.0) |  |
| Yes | 98 (33.3) | 91 (31.0) |  |
| Liver metastasis |  |  | 0.871 |
| No | 273 (92.9) | 274 (93.2) |  |
| Yes | 21 (7.1) | 20 (6.8) |  |

UL, upper lobe; ML, middle lobe; LL, low lobe; ADC, adenocarcinoma; SCC, squamous cell carcinoma; NSCLC, non-small cell lung cancer; PSM, propensity score matching

**Figure legends of the supplement figures**

**Figure S1.** The calibration curves for predicting OS in the training cohort (A, B) and the validation cohort (C, D). Nomogram-predicted survival probability is plotted on the x-axis; Actual observed survival probability is plotted on the y-axis. A curve along the 45-degree line indicates perfect calibration models. OS, overall survival.

**Figure S2.** Validation of the risk-classifying systems. (A) ROC curves comparison: Risk-classifying system A vs. TNM staging system, (B) DCA comparison: Risk-classifying system A vs. TNM staging system, (C) ROC curves comparison: Risk-classifying system B vs. M staging system and (D) DCA comparison: Risk-classifying system B vs. M staging system. ROC: receiver operating characteristic, DCA: decision curve analyses; TNM, tumor-node-metastasis
